# A Coding Basis and Three-in-One Integrated Data Visualization Method ‘Ana’ for the Rapid Analysis of Multidimensional Omics Dataset

**DOI:** 10.3390/life12111864

**Published:** 2022-11-12

**Authors:** Hefei Zhao, Selina C. Wang

**Affiliations:** Department of Food Science and Technology, University of California, Davis, One Shields Ave, Davis, CA 95616, USA

**Keywords:** multidimensional dataset, omics, 3D heatmap, hierarchical clustering analysis, principal component analysis, olive, phenolics, phytochemical, MATLAB^®^

## Abstract

With innovations and advancements in analytical instruments and computer technology, omics studies based on statistical analysis, such as phytochemical omics, oilomics/lipidomics, proteomics, metabolomics, and glycomics, are increasingly popular in the areas of food chemistry and nutrition science. However, a remaining hurdle is the labor-intensive data process because learning coding skills and software operations are usually time-consuming for researchers without coding backgrounds. A MATLAB^®^ coding basis and three-in-one integrated method, ‘Ana’, was created for data visualizations and statistical analysis in this work. The program loaded and analyzed an omics dataset from an Excel^®^ file with 7 samples * 22 compounds as an example, and output six figures for three types of data visualization, including a 3D heatmap, heatmap hierarchical clustering analysis, and principal component analysis (PCA), in 18 s on a personal computer (PC) with a Windows 10 system and in 20 s on a Mac with a MacOS Monterey system. The code is rapid and efficient to print out high-quality figures up to 150 or 300 dpi. The output figures provide enough contrast to differentiate the omics dataset by both color code and bar size adjustments per their higher or lower values, allowing the figures to be qualified for publication and presentation purposes. It provides a rapid analysis method that would liberate researchers from labor-intensive and time-consuming manual or coding basis data analysis. A coding example with proper code annotations and completed user guidance is provided for undergraduate and postgraduate students to learn coding basis statistical data analysis and to help them utilize such techniques for their future research.

## 1. Introduction

With innovations and advancements in analytical instruments and computer technology, omics studies based on statistical analysis, such as phytochemical omics [1], oilomics/lipidomics [2], proteomics [3], metabolomics [4], and glycomics [5], are increasingly popular in the areas of food chemistry and nutrition science [6,7]. Clear graphical representation and visual communication are effective ways to present large datasets and dense information to learners. Heatmaps with hierarchical clustering analysis and principal component analysis (PCA) are commonly used cluster analysis methods for omics studies. Wang et al. [8] investigated the interaction of fruity aromas with polyphenols by the use of heatmap cluster analysis by Origin Pro 9.0; Varunjikar et al. [9] analyzed proteomics from tandem mass spectrometry by the use of heatmap cluster analysis through Omics Explorer V 3.6 software for food-grade insect protein analysis; Lin et al. [10] analyzed the glycome profile of blueberry using a heatmap via R software. Yang et al. [11] combined headspace-gas chromatography-ion mobility spectrometry (HS-GC-IMS) with PCA to detect the flavor compounds of fermented soybean products by the use of a software package with a dynamic PCA plug-in. Green & Selina [12] employed both PCA and hierarchal cluster analysis without a heatmap to classify fatty acid and sterol profiles for analyzing avocado oil quality by the use of OriginPro2016 software. Zhao et al. [13] implemented PCA in R software and machine learning algorithms in Python to classify up to ten types of major edible oils based on fatty acid profiles and Raman spectra datasets; Zhao et al. [14] also applied PCA based on R software to analyze phenolic compound profiles of different cultivars of the US midwestern grapes with selenium and lithium fertilizer treatments. Richter et al. [15] used PCA and heatmap cluster analysis to analyze inductively coupled plasma mass spectrometry (ICP-MS) data in R software for identifying food authentication of German asparagus. Zou et al. [16] analyzed a multidimensional dataset of HS-SPME-GC×GC-TOFMS of coffee using ChromaTOF^®^ (ver. 5.51, LECO Corp., St. Joseph, MI, USA), ChromaTOF Tile (ver. 1.01, LECO Corp.), R version 4.0.2, and MATLAB^®^ (ver. R2019b, MathWorks, Natick, MA, USA).

However, processing high-dimensional data from raw food omics datasets is time-consuming [17,18,19] and remains a challenging task for data mining and untargeted foodomics studies [20,21]. To achieve multiple data analysis methods, different software or code packages may be needed. For instance, by the use of R software, packages ‘ggbiplot’ [22] and ‘ggplot’ [23] are usually used for PCA analysis, while another package ‘heatmap2′ [24] is usually applied for heatmap cluster analysis. However, it takes time for researchers to learn and operate different software and code packages with confidence.

The objective of the study is to develop an integrated code basis program based on MATLAB^®^ software to give a 3D heatmap, heatmap hierarchical clustering analysis, and PCA all at once by directly reading datasets from Excel^®^ files. The code has been optimized for figure qualities such as resolution, color code, and label font size. The code also adjusts the size of the 3D bars of the heatmap in accordance with the values, which gives readers better data visualization and differentiation. In addition, we have provided proper code annotations and completed user guidance in the supplementary materials for future learning and educational proposes.

## 2. Methods

### 2.1. Data Preparation

The original dataset of our previous publication about the US California olive pomace phenolics [25] was used as an example dataset in this study. As can be seen from Table S1, the data matrix contained 7 extracts * 22 olive pomace phenolic compounds. Data were saved in an ‘.xlsx’ file format by the use of Microsoft^®^ Excel; in this case, the full file name was ‘olivephenolics.xlsx’. Here, Hadley Wickham’s ‘Tidy Data’ concept [26] was referred, where each variable (22 phenolics) was a column and each sample observation (7 extracts) was a row, because the input data must be tidy for the best results. It can be seen from Figure S1a that the names of 7 olive pomace extracts were listed in the first column from A2 to A8, and the names of 22 olive pomace phenolic compounds were listed in the first row from B1 to W1. The text ‘NAME’ was placed in cell A1. The file was saved as ‘olivephenolics.xlsx’ in a MATLAB work folder.

The data area in the excel file can be expanded in both rows and columns; however, there should be no blank cells in any places in the data area. The sample observation name should also be listed in the first column and the compound variables names should be listed in the first row.

Omics data of each sample must be listed in each row, and variables/compounds must be listed in columns; otherwise, the program will still run, but output meaningless results.

The excel data file and ‘.m’ code in the MATLAB files have been uploaded to the file exchange website as a secondary way to obtain the dataset and code. Readers can download from there in the MATLAB software, as shown in the ‘screenshot’ in Figure S1b, or via the MATLAB file exchange website [27].

### 2.2. Software and Coding

MATLAB^®^ 2022a (MathWorks, Natick, MA, USA) with an academic license from the University of California, Davis (UC Davis) was used for all coding and data analysis. The ‘core’ MATLAB functions used for statistical analysis were ‘bar3′ [28], ‘clustergram’ [29] with the ‘average’ linkage as the clustering instrument, and ‘biplot’ [30] for a 3D bar heatmap, heatmap hierarchical clustering analysis, and a biplot of principal component analysis (PCA) analysis, respectively. S. Code 1 was originally designed by the authors based on those ‘core’ MATLAB functions. The bottom size adjustment of the 3D bar chart heatmap referred to the question ‘How do I obtain bars with function bar3 and different widths for each bar?’ [31] on ‘stackoverflow.com’ with modifications.

The MATLAB ‘.m’ file was prepared by the ‘copy and paste’ of S. Code 1 ‘Ana’ version 1.0 into a new ‘.m’ file window. In this case, the full file name was ‘Ana.m’, based on the description and guidance of Figure S2. Both the excel ‘olivephenolics.xlsx’ file and the MATLAB ‘Ana.m’ file was and must be saved in the same folder for successfully running the program; otherwise, the program will not run properly, because the program cannot find the excel data file if the file is in any different folder.

### 2.3. Hardware

Both Apple^®^ MacOS Monterey and Microsoft^®^ Windows 10 environments were employed for testing the code compatibility. The hardware for MacOS was a 2.3 GHz Quad-Core Intel Core i5 Processor and 8 GB 2133 MHz LPDDR3 RAM. The hardware for Windows was a 4.1 GHz 8-Core 16-Thread AMD Ryzen™ 7 2700X Processor and 16 GB 3200 MHz DDR4 RAM.

## 3. Results and Discussion

### 3.1. Heatmap 3D Bar Chart

**Figure 1:**
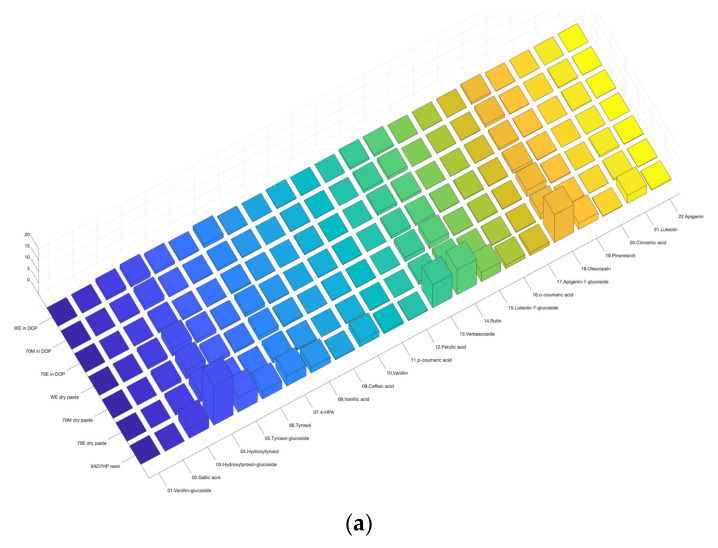

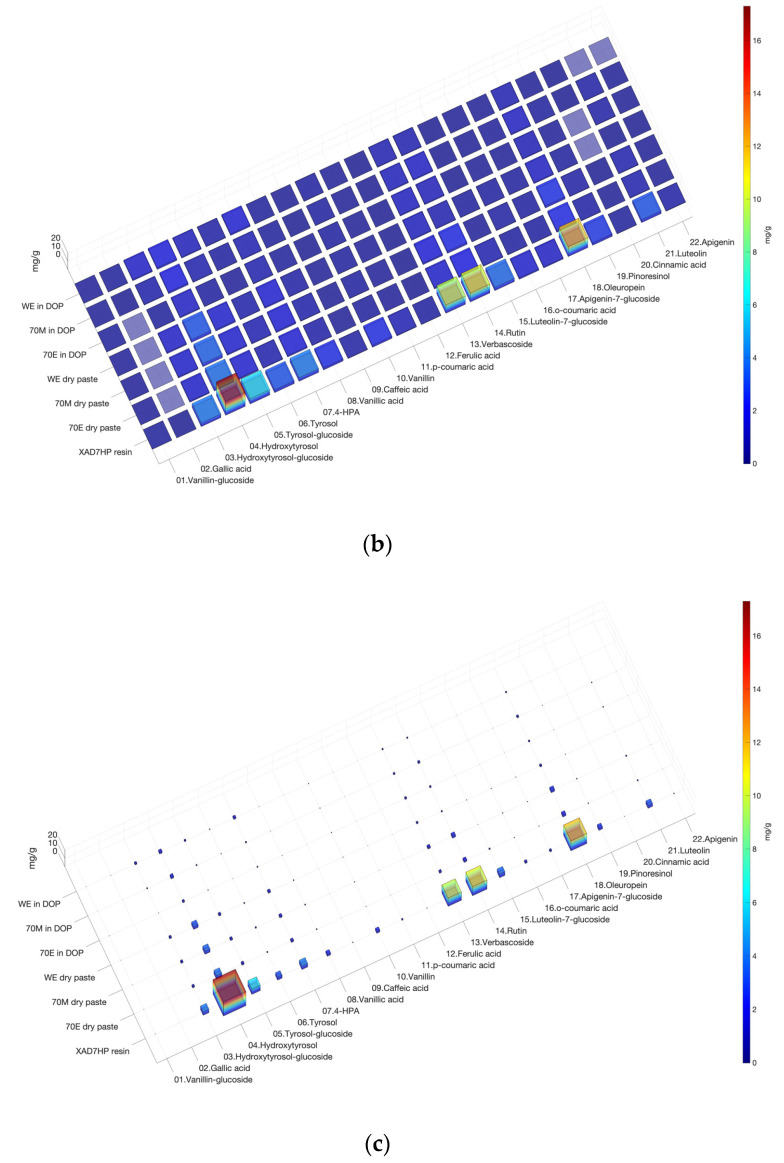
3D heatmap bar chart of olive pomace phenolic compound profile. (**a**) Figure plotted by original MATLAB code of ‘bar3′ in default colormap; (**b**) figure quality improved by adding x and y axis labels, and increasing the font size of labels; (**c**) adjusting the bottom length and width of each 3D bar according to the values that outstand the high-value data; the height and color code of each column represents the true concentrations of each phenolic, while the volume and size represent the relative and underestimated concentrations, respectively. Note: the colormap of (**b**,**c**) was rainbow by ‘jet(256)’. Please refer to Figure S3 for rotating the chart to a proper angle to display a nice visualization. WE, water extract; 70M, 70% methanol extract; 70E, 70% ethanol extract; XAD7HP resin, XAD7HP resin purified extract.

As can be seen from Figure 1a, the 3D heatmap bar chart generated by the original code ‘bar3′ would not meet the general figure quality requirement for peer-reviewed publications. The default label font size of the three axes was too small to read. The color code also differentiated compounds from blue to yellow; however, the most popular color code differentiation was based on values of compound concentrations from high to low. In addition, the bars were not transparent, which made the lower bars belied by higher bars. In general, the readability of the figure from the original code is not enough for scientific readers.

Figure 1b has been presented in our previous publication [25]. As compared with Figure 1a, the font size of labels on the three axes was enlarged for better readability. In addition, a color code bar was added to represent higher values in red and lower values in blue. The figure was printed in high resolution at 300 dpi. However, the major problem is that the lower values in blue almost dominated the entire chart and could not be easily differentiated. An interesting conversation [31] on ‘stackoverflow.com’ described a method to resize the bottom length and width based on the values in each data cell. The idea is to increase the size of the bottom when the value is higher while decreasing the size of the bottom when the value is lower. By incorporating the idea and code modifications, the minor compounds did not dominate the screen and the readability increased in Figure 1c.

**Figure 2:**
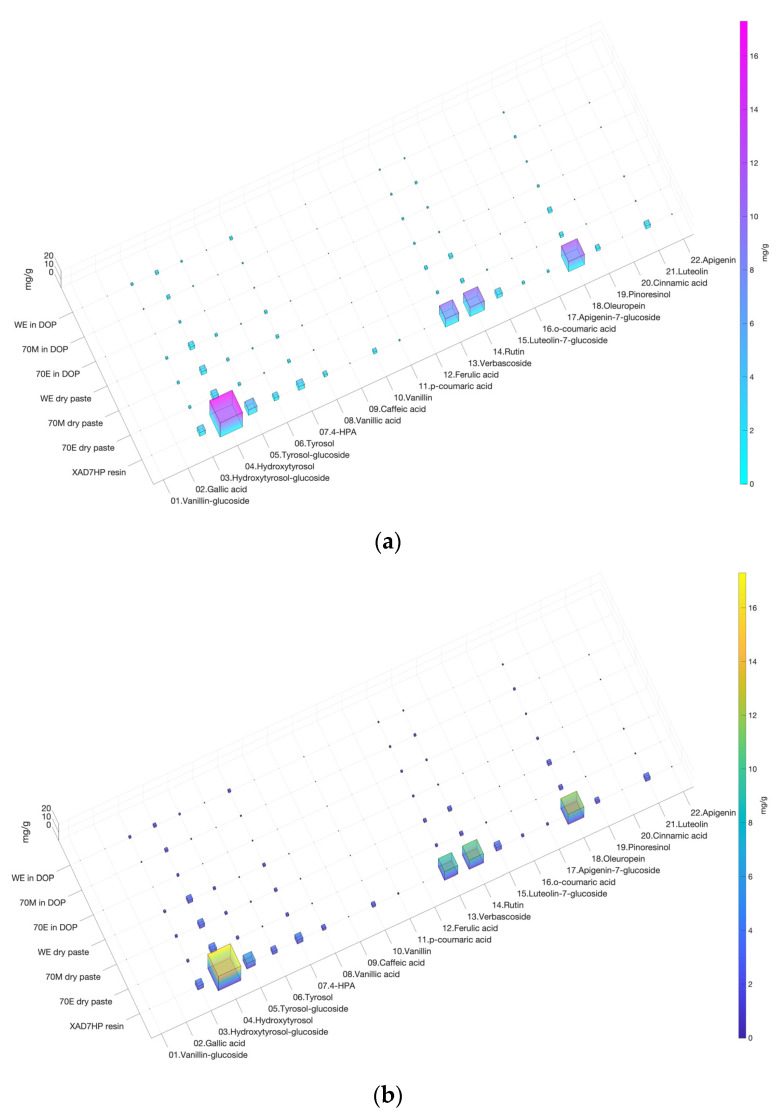

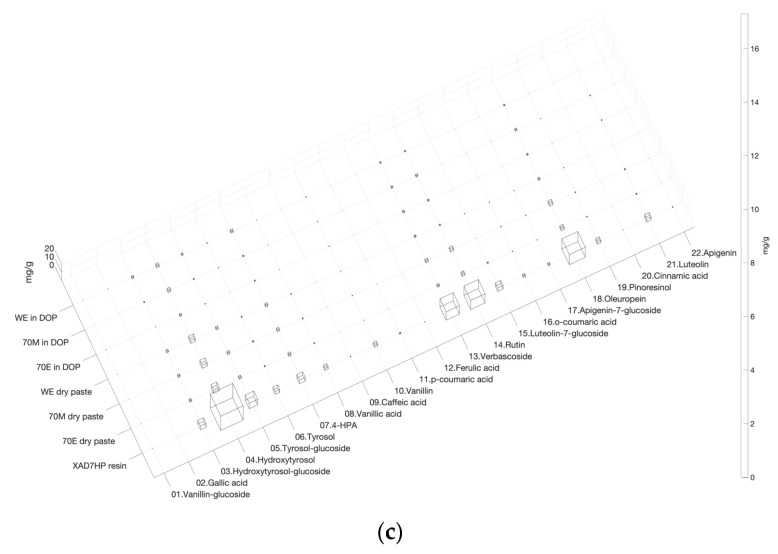
Different colormap comparisons of 3D heatmap bar charts. (**a**) Colormap ‘cool’ from blue to pink; (**b**) colormap ‘parula’ from blue to yellow; (**c**) ‘[]’ transparent.

In addition, S. Code 1 provides different options of color schemes as can be seen in Figure 1c and Figure 2. In the S. Code1, jet(256) outputs rainbow in Figure 1c; cool is blue to pink in Figure 2a; parula is blue to yellow in Figure 2b; ‘[]’ is transparent in Figure 2c. The code also provides different resolution options from 100 to 300 dpi. The output figures were rich in color and provided enough contrast in both color and bar size to differentiate the omics dataset.

### 3.2. Heatmap Cluster Analysis

**Figure 3:**
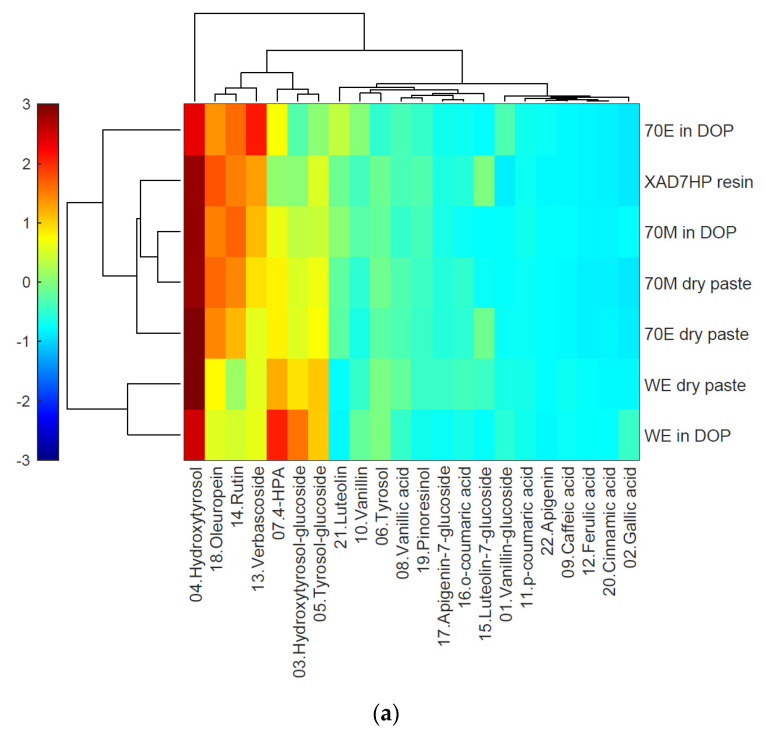

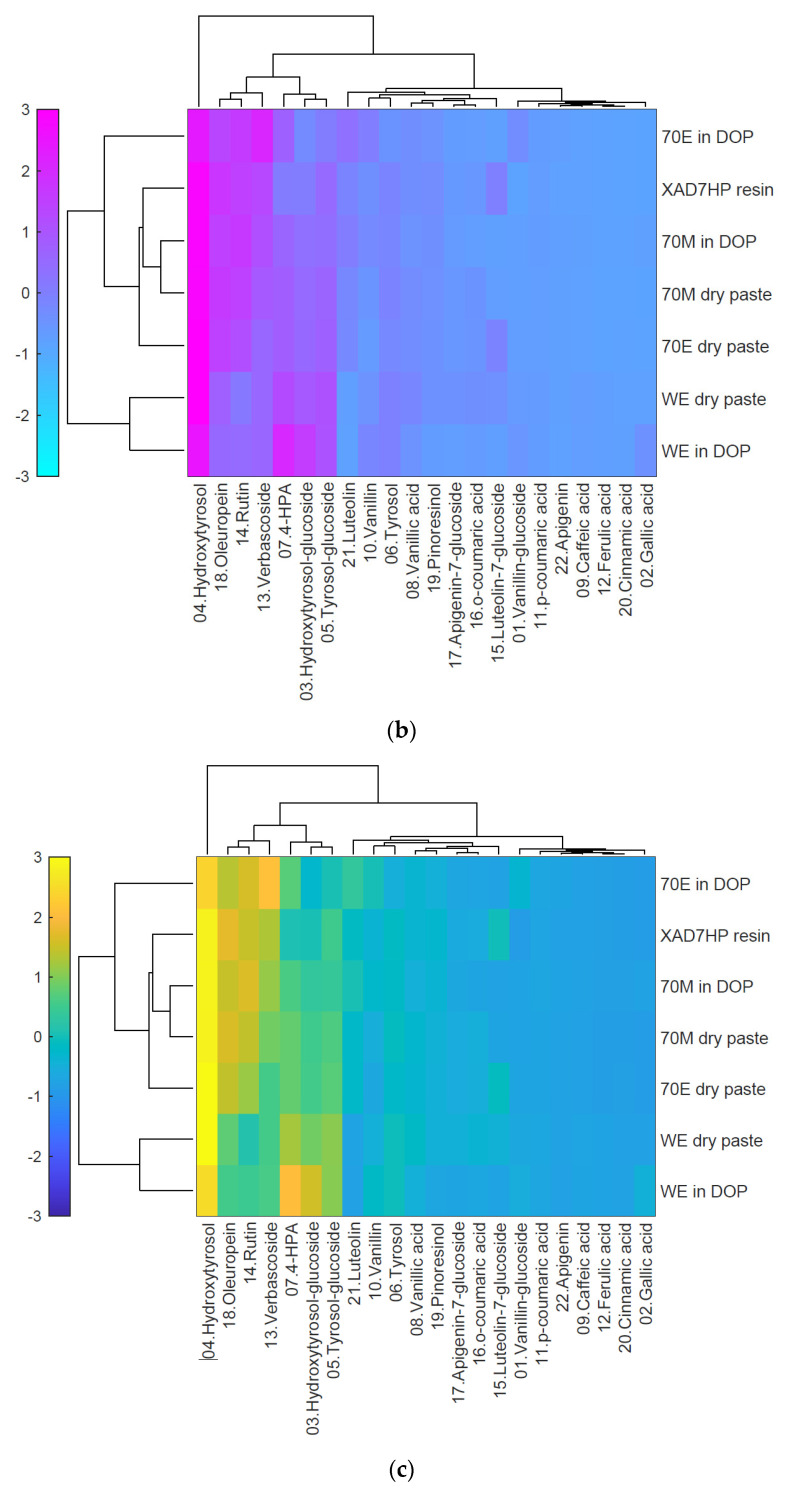

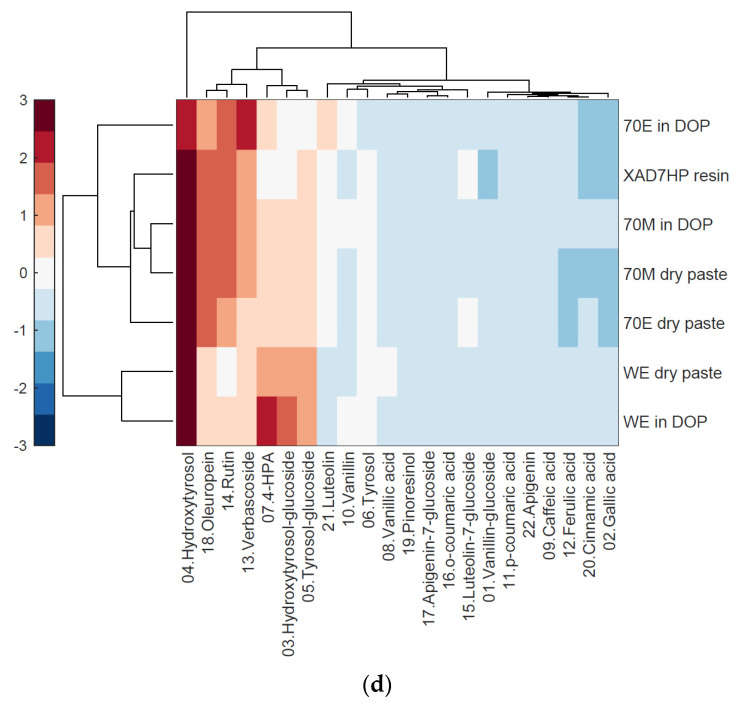
Heatmap hierarchical clustering analysis; data were standardized on each row for comparison of the profile, (**a**) colormap ‘jet(256)’ for rainbow; (**b**) colormap ‘cool’ from blue to pink; (**c**) colormap ‘parula’ from blue to yellow; (**d**) colormap ‘redbluecmap’ from blue to red.

With high data density and revealing clusters, heatmap hierarchical clustering analysis provides better visualization than unordered heatmaps [32]. Because the program standardizes the data along each sample row, the row cluster on the left side in Figure 3 grouped the samples based on olive phenolic compound profiles instead of absolute values. The samples WE dry past and WE in DOP formed one cluster, indicating that they had more similarity than the other samples, such as 70M and 70E. The program also provides options for different color codes as can be seen in Figure 3a–d.

### 3.3. PCA Analysis

**Figure 4:**
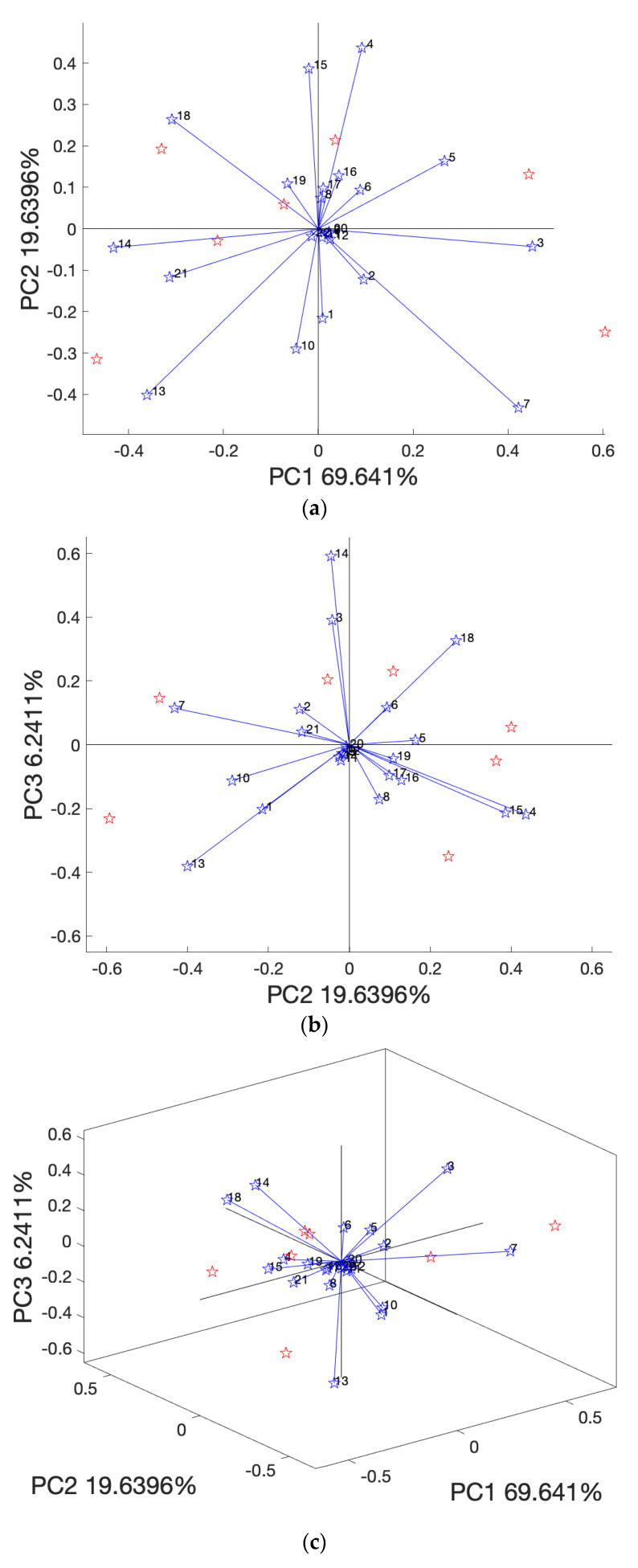

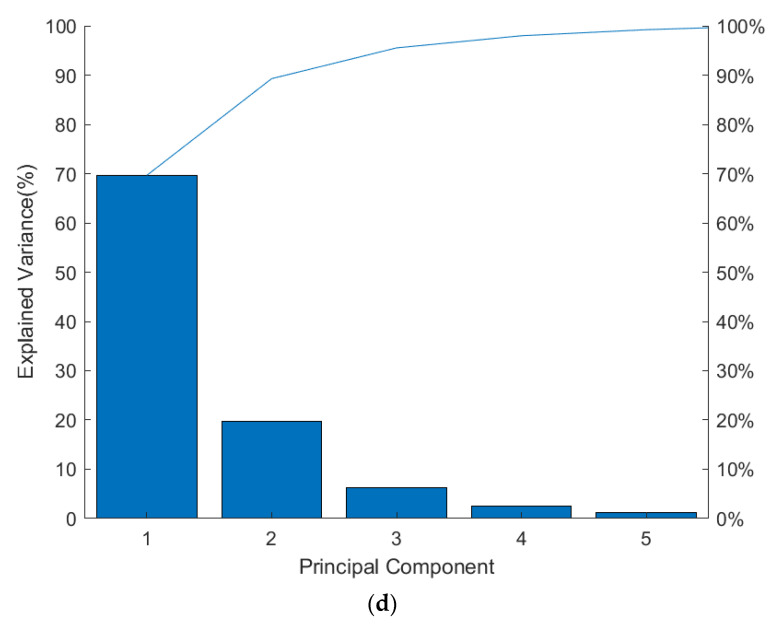
Principal component analysis (PCA) biplot, (**a**) biplot of PC1 vs. PC2, (**b**) biplot of PC2 vs. PC3, (**c**) biplot of PC1 vs. PC2 vs. PC3, and (**d**) explained variance; bars represent the variance of individual PCs and the line represents accumulated variances. Note: Blue stars 1–22 in (**a**–**c**) represent variable/phenolic compound names (the first row) in Table S1. Red stars are the seven samples.

**Figure 5:**
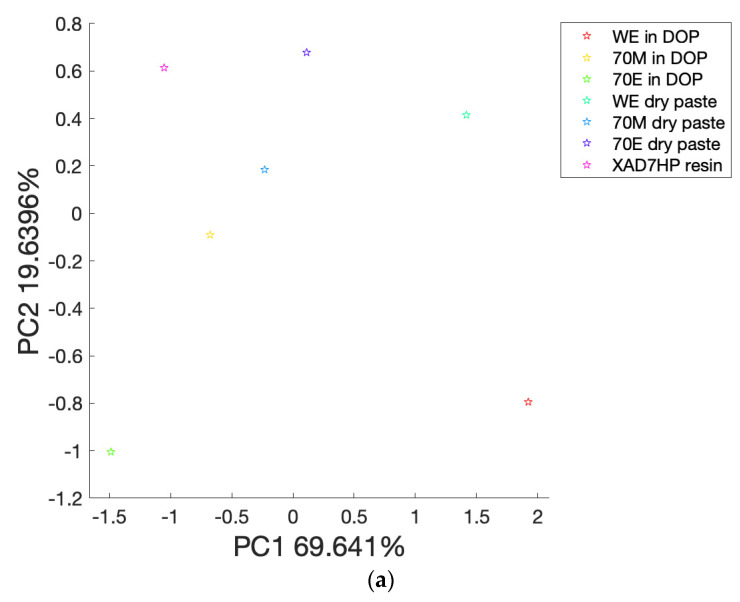

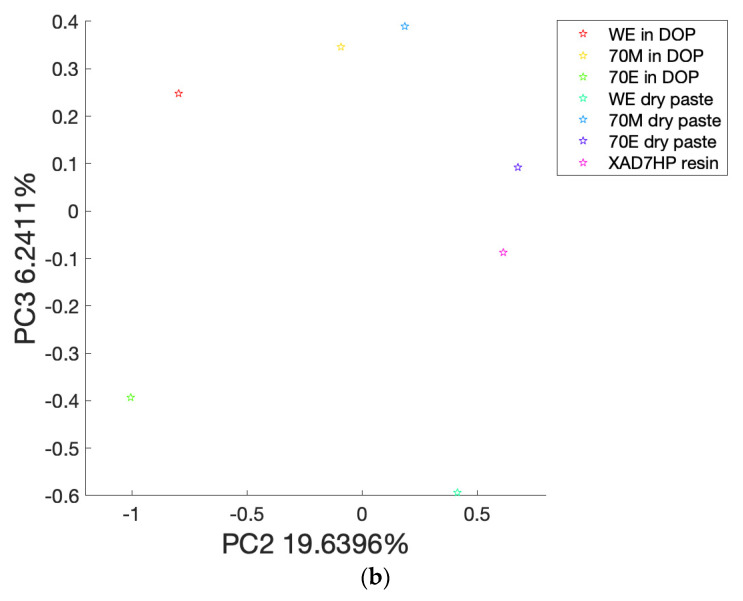
Principal component analysis (PCA) score plot: (**a**) score plot of PC1 vs. PC2; (**b**) score plot of PC2 vs. PC3.

PCA is a dimensional reduction statistical analysis method that can be implemented to reduce the dimension of original variables to several top principal components (PCs) with most of the explained variances [33]. As shown in Figure 4a–c, the PCA biplot printed PC1 vs. PC2, PC2 vs. PC3, and PC1 vs. PC2 vs. PC3, respectively. The PCA biplot overlays the loading plot (blue vectors) and the score plot (red starts) on the one graph [34,35]. The vectors of loading plots represent the multivariate variables (in this case, the olive phenolic compounds in Table S1) that affect the differences among samples [36]. The score plot shows dot points (red starts) that represent the original samples [33]. However, the PCA biplot did not differentiate samples by different colors or dot styles. Therefore, the program ‘Ana’ was designed to output separated score plots in Figure 5 by the use of different colors for samples. Figure 4d outputs the variance of individual PCs until 98% accumulated variances.

The PCA analysis here integrated into ‘Ana’ version 1.0 has yet to differentiate sample clusters (i.e., replicated or triplicated data for each sample) by the use of both different dot styles: color codes such as the PCA score plots in the work of Zhao et al. [37] for Raman spectra of egg white protein analysis by R software, and the 95% confidence eclipse in the plot work of Uchimiya [38] for the resistant genotype and underlying chemistry of sweet sorghum juice. Nevertheless, updated versions of the program would be expected to include those functions in the future.

### 3.4. Time Taking and Code Compatibility

As shown in Figure S5, the program analyzed the dataset with 7 samples * 22 compounds and output six figures for three types of data visualization, including a 3D heatmap, heatmap hierarchical clustering analysis, and principal component analysis (PCA), respectively, in 18 s on a personal computer (PC) with a Windows 10 system and in 20 s on a Mac with a MacOS Monterey system. The code basis analysis is rapid and compatible with the two different operating systems.

## 4. Conclusions

The improved MATLAB^®^ coding basis data analysis and visualization method, ‘Ana’ version 1.0, outputs three types of data analysis, including a 3D heatmap, heatmap hierarchical clustering analysis, and PCA, by one program running in seconds. The code is rapid and efficient to print out high-quality figures up to 150 or 300 dpi. The colored output figures provide enough contrast to differentiate the omics dataset by both color code and bar size difference, allowing the figures to be qualified for publication and presentation purposes. The program is compatible with both Windows and MacOS operating systems.

With completed guidance in the Supplementary Materials, the analysis program would liberate researchers from labor-intensive and time-consuming manual or coding basis data analysis and would enable them to fully focus on the results of their specific area of research with a single click of the ‘Run’ button on the software. This study also provides a coding example with appropriate code annotations for undergraduate and postgraduate students to learn coding basis statistical data analysis and to help them utilize such techniques for their future research.

## Data Availability

All the data and MATLB code have been provided in the Supplementary Materials.

## Supplementary Materials

The following supporting information can be downloaded at: https://www.mdpi.com/article/10.3390/life12111864/s1. Figure S1: Data and code preparation; Figure S2: MATLAB .m file preparation; Figure S3: By clicking the rotation icon (red arrow) in (a), figures will pop up, then (b) copy print(fig3Dbar,’olive-adjudtsize.png’,’-dpng’,’-r150’), then paste in the command window (the blue arrow), then press keyboard ‘Enter’, a ‘.png’ file at 150 dpi will show up in the ‘current folder’ (red arrow); Figure S4: Export heatmap cluster chart to a ‘.pdf’ file, (a) on the ‘Clustergram 1′ window, click ‘Insert Colorbar’, (b) on the ‘Clustergram 1′ window, click ‘File’, then click ‘Export Setup’, (c) click ‘Rendering’, select ‘Custom rendering’ as ‘Painters (vector format)’, very important for high-resolution output!!!, click ‘Apply to Figure’, then click ‘Export’, (d) input ‘File name’ as ‘Cluster’, select ‘Save as type’ the ‘Portable Document Format (*.pdf)’, (e) click ‘Save’, (f) a ‘Cluster.pdf’ file will show up in the ‘Current Folder’. Then open the ‘.pdf’ file for ‘print screen’ a high-resolution figure; Figure S5: Final outcomes of the program running; Table S1: Phenolic compound data of olive pomace extract, data from our previous publication.
