# Supplementary material for: A Coding Basis and Three-in-One Integrated Data Visualization Method ‘Ana’ for the Rapid Analysis of Multidimensional Omics Dataset
Source: Life (Basel). 2022 Nov 12;12(11):1864. doi: 10.3390/life12111864 (PMC9698950; doi:10.3390/life12111864)
Supplement: Supplementary file 1 [file life-12-01864-s001.zip › life-1986679-supplementary.pdf]

Supplementary materials

A Coding Basis and Three-in-One Integrated Data Visualization Method 'Ana' for the Rapid Analysis of Multidimensional Omics Dataset

Hefei Zhao and Selina C. Wang *

Department of Food Science and Technology, University of California, Davis, One Shields Ave, Davis, CA 95616, USA

*Corresponding: scwang@ucdavis.edu

S. Codes

S. Code 1 Save the following codes between the two lines in the 'Ana.m' file in MATLAB as shown in S. Fig 2

```
%---------------------
% 09/02/2022 the .m file 'Ana' version 1.0 was created by Hefei Zhao, PhD. This code is designed for analyzing olive pomace polyphenols concentrations from HPLC-DAD as well as many other omics data.

% Supplementary materials. S. Code 1
% A coding basis and three-in-one integrated data visualization method 'Ana' for the rapid analysis of multidimensional omics dataset
% Hefei Zhao1, Selina C. Wang1,*
% 1Department of Food Science and Technology, University of California, Davis, One Shields Ave, Davis, CA 95616, USA
% *Corresponding author: Selina C. Wang, email: scwang@ucdavis.edu
% First author: Hefei Zhao, email: hzhao@huskers.unl.edu; hefzhao@ucdavis.edu
%
% References
% Zhao, H., Avena-Bustillos, R. J., & Wang, S. C. (2022). Extraction, Purification and In Vitro Antioxidant Activity Evaluation of Phenolic Compounds in California Olive Pomace. In Foods (Vol. 11, Issue 2). https://doi.org/10.3390/foods11020174
% matlab - How I obtain bars with function bar3 and different widths for each bar? - Stack Overflow. (n.d.). Retrieved September 3, 2022, from https://stackoverflow.com/questions/24269516/how-i-obtain-bars-with-function-bar3-and-different-widths-for-each-bar

% %%%%%%%%%%%%%%%%%%%%%%%%%%%%%%%%%%%
% The percentage icon '%' means the contents after the % are all text notes instead of executable code.
clear, clc% clean RAM and command window
close all% close all figures, but Clustergram 1 must be closed manully.
tic % start timing
%%%%%%%%%%%%%%%%%%
% Initial input area for normal users or beginners

fn= 'olivephenolics';% input the excel file name in the '' area, olivephenolics can be replaced by user's excel file name, the excel file must be in the same folder as the this .m file
unt= 'mg/g';% input unit of data in the '' area, mg/g can be replaced by the user's unit, such as %, g/mL, mg/mL etc.
fs= 20;% input font size, 18-22 are recommended, must be >=7
cl= jet(256);% color style: jet(256) is rainbow; cool is blue to pink;  parula is blue to yellow; redbluecmap is blue to red; [] is transparent; user can replaced jet(256) by cool, [] or parula or bredbluecmap, etc.
pcamz= 10;% PCA marker size
pcalable= 0;% 0 will lable PCA vectors by variable/ compound numbers; 1 will lable PCA vectors by variable/ compound full names.
mk= '.'; % set PCA data marker in '' area; . is dot; p is star; s is square; * is snow flasker; o is o; 'd' is rhombus.

% Note: Method for print or output figures:
```

```
% Figure 1, 3D heatmap
% print(fig3Dbar,'olive-adjudtsize.png','-dpng','-r150');% -r150 defines 150dpi,-r300 will provide 300 dpi, -
r100 will provide 100 dpi, etc. See S. Fig. 3

% Clustergram 1, heatmap cluster, can only be output to a pdf for high-resolution as descripted in S. Fig.
4.

% Figure 2-7, print PCA charts automatically at 150 dpi

%%%%%%%%%%%%%%%%%%%
% It may result in errors if modify code below this line if not skillful on MATLAB.
% But it would be greatly helpful for active learners to modify the code to improve coding skills.

fname= strcat(fn);% excel file name
info= readtable(fname,'PreserveVariableNames',true, 'ReadRowNames',true);% read data again for
column names
rm= string(info.Properties.RowNames);% read row names of samples
cm= string(info.Properties.VariableNames);% read column/variable names of phenolic compounds
sz= size(info);% read data area size
ns= sz(1);% read sample number
nb= sz(2);% read compound number
num= readmatrix(fname); % read data only
num(:,1) = []; % delete first column NAN
ave= num; % define ave values
%

% plot 3D bar chart with size adjustments
%
fig3Dbar=figure; % name figure as fig3Dbar
h=bar3(ave,'detached');%'detached', 'grouped', or 'stacked'
%
values=ave; % values equal to ave.
m= 1.1*max(values(:))*2; % normalize constant for bar width, 1.1 ensure a small distance between bars
shading interp % set color shading properties
for i = 1:length(h)% the following code set bottom size in accordance with the z-axis values
    % Get the ZData matrix of the current group
    xdata= get(h(i),'Xdata');
    ydata= get(h(i),'Ydata');
    zdata= get(h(i),'Zdata');
    set(h(i),'Cdata',zdata)
    for k = 1:size(xdata,1)/6
        datax = xdata((k-1)*6+1+(0:5),:);
        datay = ydata((k-1)*6+1+(0:5),:);
        dirx=((datax-round(datax))>0)-((datax-round(datax))<0);
        diry=((datay-round(datay))>0)-((datay-round(datay))<0);
        xdata((k-1)*6+1+(0:5),:) = round(xdata((k-1)*6+1+(0:5),:),0)+dirx*values(ceil(((k-1)*6+1)/6),i)/m;
        ydata((k-1)*6+1+(0:5),:) = round(ydata((k-1)*6+1+(0:5),:),0)+diry*values(ceil(((k-1)*6+1)/6),i)/m;
    end
```

```
    set(h(i),'XData',xdata);
    set(h(i),'YData',ydata);
end
set(h,'EdgeColor','k') % set edge color as k black
view(-25, 83); % default view angle
colormap (cl) % set colormap as cl
colorbar % show color bar
%
xticks(1:nb)% add x ticks
yticks(1:ns)% add y ticks
set(gca,'XTickLabel',cm)% label x axis by column/variable names of phenolic compound
set(gca,'YTickLabel',rm)% label y axis by row names of samples
set(h,'FaceAlpha',.5) % set transparency of bars to 0.5
zlabel(unt,'FontSize',fs) % set font size of z-axis
ax = gca;
ax.FontSize = fs; % set font size of color code bar
cb=colorbar;
colormap(cl); % define color as descripted by cl
cb.Label.String = unt; % set unit of color code bar as descripted by unt
%print(fig3Dbar,'olive-adjudtsize.png','-dpng','-r150');% -r150 defines 150dpi,-r300 will provide 300 dpi, -
r100 will provide 100 dpi, etc.

% Cluster analysis
cfac=clustergram(ave, 'RowLabels',
rm,'ColumnLabels',cm,'Colormap',colormap(cl),'Standardize','Row'); % the code standardize data on each
row
% For more information see: https://www.mathworks.com/help/bioinfo/ref/clustergram.html
set(cfac,'Linkage','complete','Dendrogram',10)
set(cfac,'Annotate','off') % turn of annotate
set(cfac,'Linkage','Average') % set linkage method by Average
set(cfac,'RowPDist','Euclidean') % row distance method Euclidean
set(cfac,'ColumnPDist','Euclidean') % column distance method Euclidean
%
% PCA
sdz = zscore(ave,[],2); % standardized data along data rows
[coefs,score,latent,tsquared,explainedvariance] = pca(sdz); % run PCA
%
if pcalable== 0 % 0 will lable PCA vectors by variable/ compound numbers; 1 will lable PCA vectors by
variable/ compound full names.
    lbls= string(1:length(cm));
else
    lbls= cm;
end
%
figPCA12biplot= figure; % open a new figure
pa12= biplot(coefs(:,1:2),'Scores',score(:,1:2),'VarLabels',lbls,'Marker',mk,'MarkerSize',pcamz); % plot
PCA biplot of PC1 vs. PC2
grid off % turn off grid
```

```
box off % ture off box
ax = gca;
ax.FontSize = fs-6; % set font size of tick, 6 less than label font size
xlabel(['PC1',' ', num2str(explainedvariance(1)),'%'],'FontSize',fs)% label x-axis
ylabel(['PC2',' ', num2str(explainedvariance(2)),'%'],'FontSize',fs)% label y-axis
print(figPCA12biplot,'PCA12-biplot.png','-dpng','-r150');% print figure at 150 dpi
%
clr = hsv(ns);
figPCA12score= figure;
gscatter(score(:,1),score(:,2),rm,clr,mk) % plot PCA scoreplot of PC1 vs. PC2
legend('Location','northeastoutside')
grid off % turn off grid
box off % ture off box
ax = gca;
ax.FontSize = fs-6; % set font size of tick, 6 less than label font size
xlabel(['PC1',' ', num2str(explainedvariance(1)),'%'],'FontSize',fs)% label x-axis
ylabel(['PC2',' ', num2str(explainedvariance(2)),'%'],'FontSize',fs)% label y-axis
print(figPCA12score,'PCA12-score.png','-dpng','-r150');% print figure at 150 dpi
%
figPCA23biplot= figure; % open a new figure
pa23= biplot(coefs(:,2:3),'Scores',score(:,2:3),'VarLabels',lbls,'Marker',mk,'MarkerSize',pcamz); % plot
PCA biplot of PC2 vs. PC3
grid off % turn off grid
box off % ture off box
ax = gca;
ax.FontSize = fs-6; % set font size of tick, 6 less than label font size
xlabel(['PC2',' ', num2str(explainedvariance(2)),'%'],'FontSize',fs)
ylabel(['PC3',' ', num2str(explainedvariance(3)),'%'],'FontSize',fs)
print(figPCA23biplot,'PCA23-biplot.png','-dpng','-r150');
%
figPCA12score= figure;
gscatter(score(:,2),score(:,3),rm,clr,mk)% plot PCA scoreplot of PC2 vs. PC3
legend('Location','northeastoutside')
grid off % turn off grid
box off % ture off box
ax = gca;
ax.FontSize = fs-6; % set font size of tick, 6 less than label font size
xlabel(['PC2',' ', num2str(explainedvariance(2)),'%'],'FontSize',fs)% label x-axis
ylabel(['PC3',' ', num2str(explainedvariance(3)),'%'],'FontSize',fs)% label y-axis
print(figPCA12score,'PCA23-score.png','-dpng','-r150');% print figure at 150 dpi
%
figPCA123biplot= figure; % open a new figure
pa123= biplot(coefs(:,1:3),'Scores',score(:,1:3),'VarLabels',lbls,'Marker',mk,'MarkerSize',pcamz); % plot
PCA biplot of PC1 vs. PC2 vs. PC3
grid off % turn off the grid
ax = gca;
ax.FontSize = fs-6; % set font size of tick, 6 less than label font size
xlabel(['PC1',' ', num2str(explainedvariance(1)),'%'],'FontSize',fs)
```

```
ylabel(['PC2',' ', num2str(explainedvariance(2)),'%'],'FontSize',fs)
zlabel(['PC3',' ', num2str(explainedvariance(3)),'%'],'FontSize',fs)% label z-axis
box on % ture on box
print(figPCA123biplot,'PCA123-biplot.png','-dpng','-r150');
%
ev= figure;
pareto(explainedvariance,0.99) % draw Explained Variance
ax = gca;
ax.FontSize = fs-6; % set font size of tick, 6 less than label font size
xlabel('Principal Component','FontSize',fs)
ylabel('Explained Variance(%)','FontSize',fs)
box off % ture off box
print(ev,'PCA-Explained-Variance.png','-dpng','-r150');
toc% stop timing

%--------------------
```

S. Figures

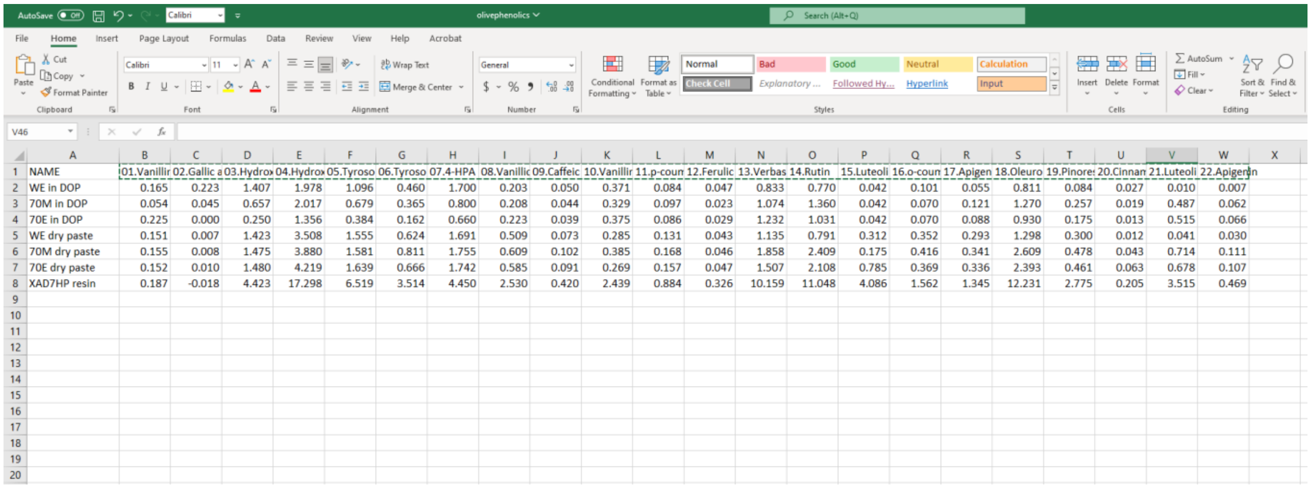

	A	B	C	D	E	F	G	H	I	J	K	L
1	NAME	01.Vanillir	02.Gallic a	03.Hydrox	04.Hydrox	05.Tyroso	06.Tyroso	07.4-HPA	08.Vanillic	09.Caffeic	10.Vanillir	11.p-coum
2	WE in DOP	0.165	0.223	1.407	1.978	1.096	0.460	1.700	0.203	0.050	0.371	0.084
3	70M in DOP	0.054	0.045	0.657	2.017	0.679	0.365	0.800	0.208	0.044	0.329	0.097
4	70E in DOP	0.225	0.000	0.250	1.356	0.384	0.162	0.660	0.223	0.039	0.375	0.086
5	WE dry paste	0.151	0.007	1.423	3.508	1.555	0.624	1.691	0.509	0.073	0.285	0.131
6	70M dry paste	0.155	0.008	1.475	3.880	1.581	0.811	1.755	0.609	0.102	0.385	0.168
7	70E dry paste	0.152	0.010	1.480	4.219	1.639	0.666	1.742	0.585	0.091	0.269	0.157
8	XAD7HP resin	0.187	-0.018	4.423	17.298	6.519	3.514	4.450	2.530	0.420	2.439	0.884

	A	M	N	O	P	Q	R	S	T	U	V	W
1	NAME	12.Ferulic	13.Verbas	14.Rutin	15.Luteoli	16.o-coun	17.Apigen	18.Oleuro	19.Pinores	20.Cinnam	21.Luteoli	22.Apigenin
2	WE in DOP	0.047	0.833	0.770	0.042	0.101	0.055	0.811	0.084	0.027	0.010	0.007
3	70M in DOP	0.023	1.074	1.360	0.042	0.070	0.121	1.270	0.257	0.019	0.487	0.062
4	70E in DOP	0.029	1.232	1.031	0.042	0.070	0.088	0.930	0.175	0.013	0.515	0.066
5	WE dry paste	0.043	1.135	0.791	0.312	0.352	0.293	1.298	0.300	0.012	0.041	0.030
6	70M dry paste	0.046	1.858	2.409	0.175	0.416	0.341	2.609	0.478	0.043	0.714	0.111
7	70E dry paste	0.047	1.507	2.108	0.785	0.369	0.336	2.393	0.461	0.063	0.678	0.107
8	XAD7HP resin	0.326	10.159	11.048	4.086	1.562	1.345	12.231	2.775	0.205	3.515	0.469

(a)

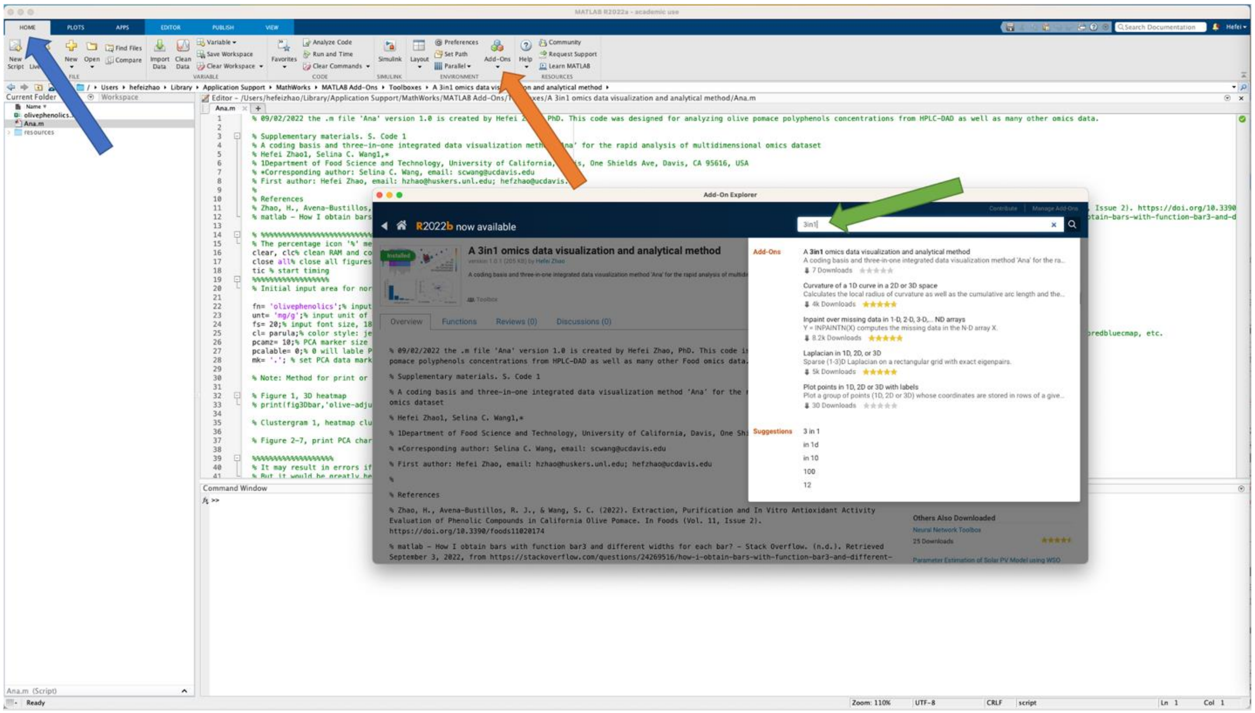

(b)

Figure S1. Data and code preparation. (a) Data from S. Table 1 was prepared in an excel file for reading from MATLAB, with one sample in each row, and one compound in each column. The 'NAME' in the cell of the first row and first column must be lined up at cell A1 in the excel file. Save as file 'olivephenolics.xlsx' in a folder. (b) Installation of data file and .m code from MATLAB 'Add-Ons': press 'HOME' tab at upper left, then click 'Add-Ons' tab, then search '3in1 omics data visualization and analytical method', then select the first result and install it.

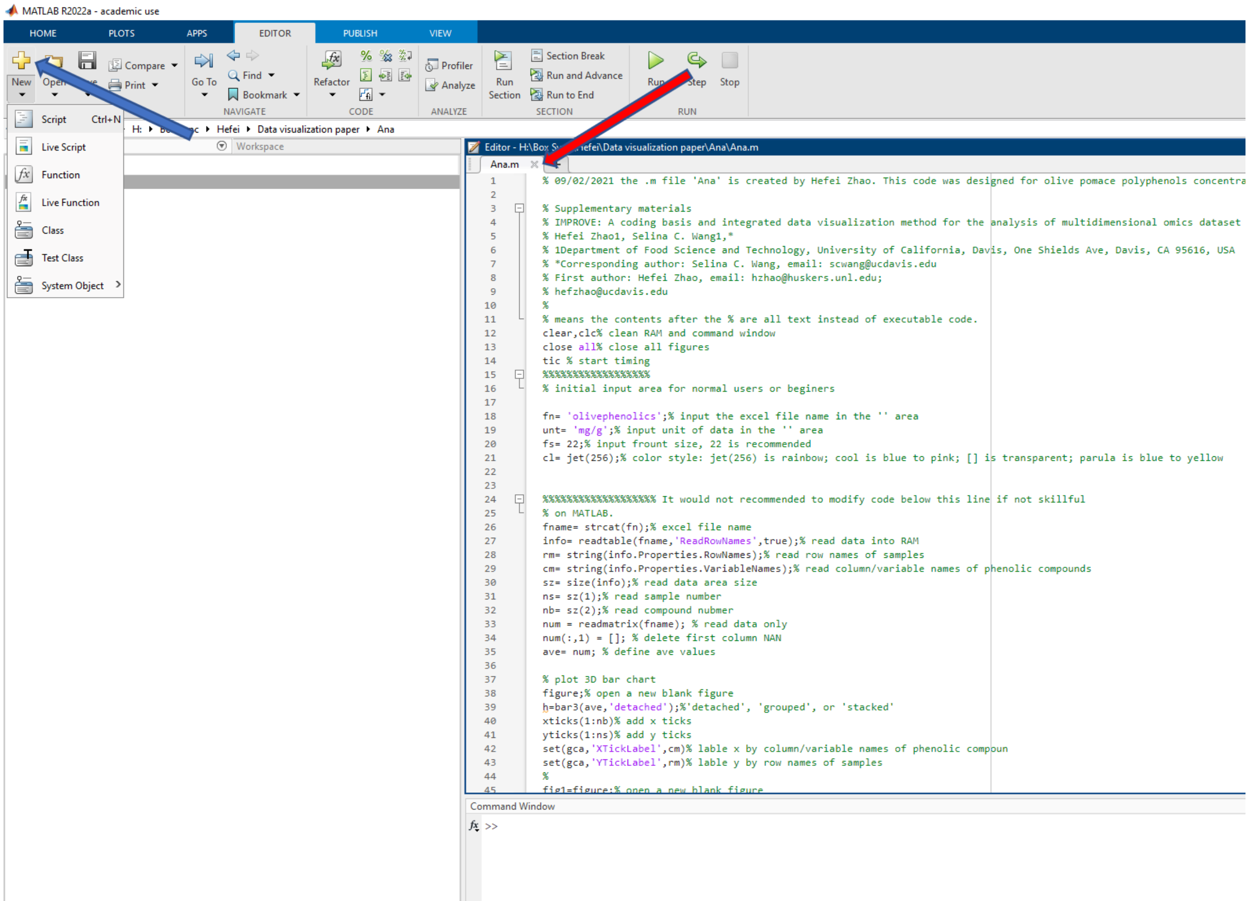
MATLAB R2022a - academic use
HOME
PLOTS
APPS
EDITOR
PUBLISH
VIEW
New
Compare
Print
Go To
Find
Bookmark
NAVIGATE
Refactor
CODE
Profiler
Analyze
ANALYZE
Run Section
Section Break
Run and Advance
Run to End
SECTION
Step
Stop
RUN
Script Ctrl+N
Live Script
Function
Live Function
Class
Test Class
System Object
Hefei
Data visualization paper
Ana
Workspace
Ana.m
Command Window
% 09/02/2021 the .m file 'Ana' is created by Hefei Zhao. This code was designed for olive pomace polyphenols concentra
% Supplementary materials
% IMPROVE: A coding basis and integrated data visualization method for the analysis of multidimensional omics dataset
% Hefei Zhao1, Selina C. Wang1,*
% 1Department of Food Science and Technology, University of California, Davis, One Shields Ave, Davis, CA 95616, USA
% *Corresponding author: Selina C. Wang, email: scwang@ucdavis.edu
% First author: Hefei Zhao, email: hzhao@huskers.unl.edu;
% hefzhao@ucdavis.edu
%
% means the contents after the % are all text instead of executable code.
clear,clc% clean RAM and command window
close all% close all figures
tic % start timing
%%%%%%%%%%%%%%%%%%
% initial input area for normal users or beginers
fn= 'olivephenolics';% input the excel file name in the '' area
unt= 'mg/g';% input unit of data in the '' area
fs= 22;% input frount size, 22 is recommended
cl= jet(256);% color style: jet(256) is rainbow; cool is blue to pink; [] is transparent; parula is blue to yellow
%%%%%%%%%%%%%%%%%%% It would not recommended to modify code below this line if not skillful
% on MATLAB.
fname= strcat(fn);% excel file name
info= readtable(fname,'ReadRowNames',true);% read data into RAM
rm= string(info.Properties.RowNames);% read row names of samples
cm= string(info.Properties.VariableNames);% read column/variable names of phenolic compounds
sz= size(info);% read data area size
ns= sz(1);% read sample number
nb= sz(2);% read compound nubmer
num = readmatrix(fname); % read data only
num(:,1) = []; % delete first column NAN
ave= num; % define ave values
% plot 3D bar chart
figure;% open a new blank figure
h=bar3(ave,'detached');%'detached', 'grouped', or 'stacked'
xticks(1:nb)% add x ticks
yticks(1:ns)% add y ticks
set(gca,'XTickLabel',cm)% lable x by column/variable names of phenolic compoun
set(gca,'YTickLabel',rm)% lable y by row names of samples
%
fig1=figure;% open a new blank figure
>>

(a)

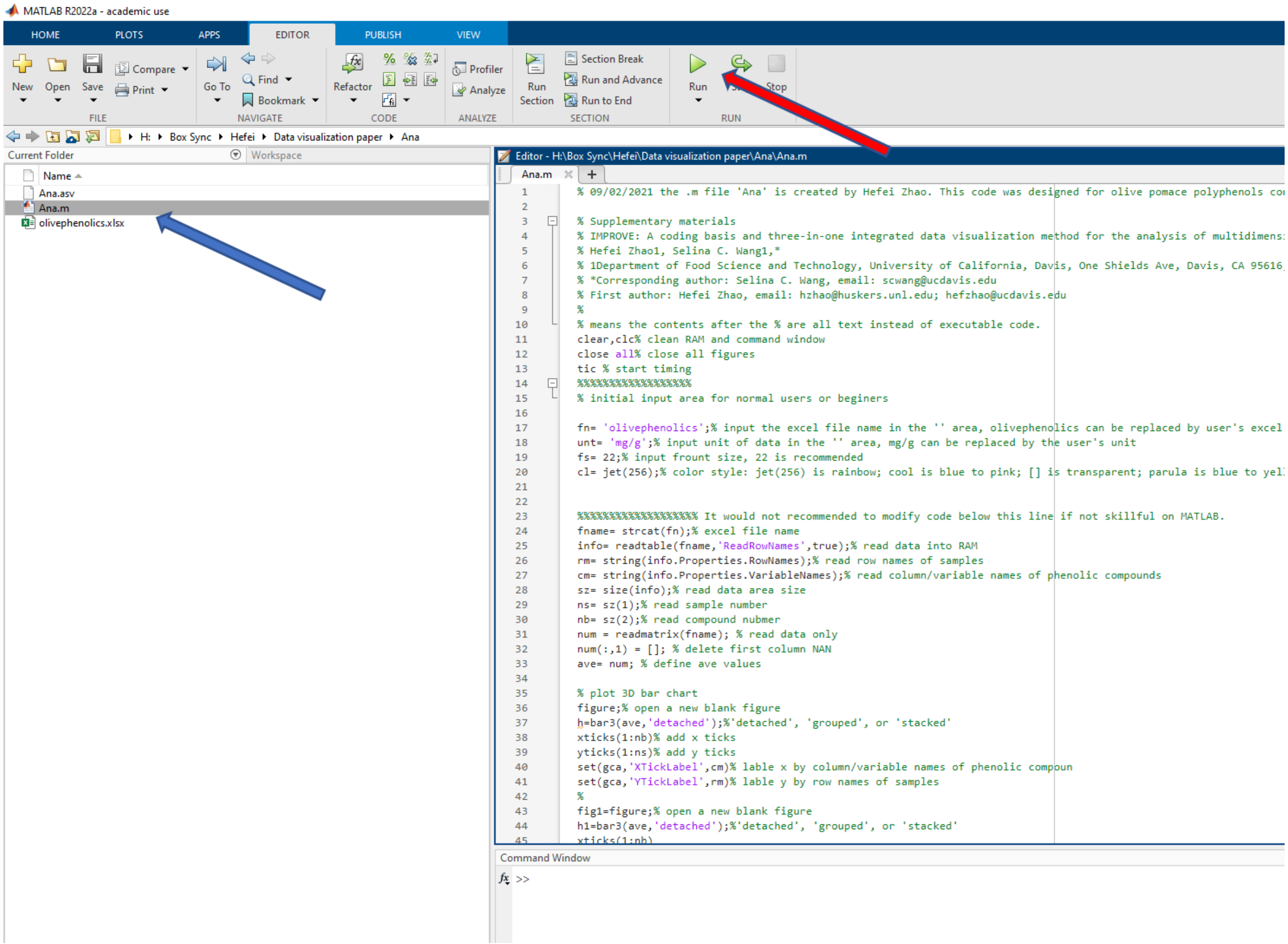

(b)

Figure S2. MATLAB .m file preparation. (a) click icon '+new' (blue arrow) at the upper left of the software interface, then copy and paste the code **S. Code 1** into the editor window (red arrow), then save the Ana.m file in the same folder as the 'olivephenolics.xlsx' file; (b) both the excel 'olivephenolics.xlsx' file and the MATLAB 'Ana.m' file must be in the same folder; normally a '.asv' file will be generated once click 'run' button to get results and figures

Note: Beside the Excel date and MATLAB '.m' files can be reconstructed from the supplementary materials, the tool packages including all necessary files can also be downloaded from MATLAB file exchange website [1].

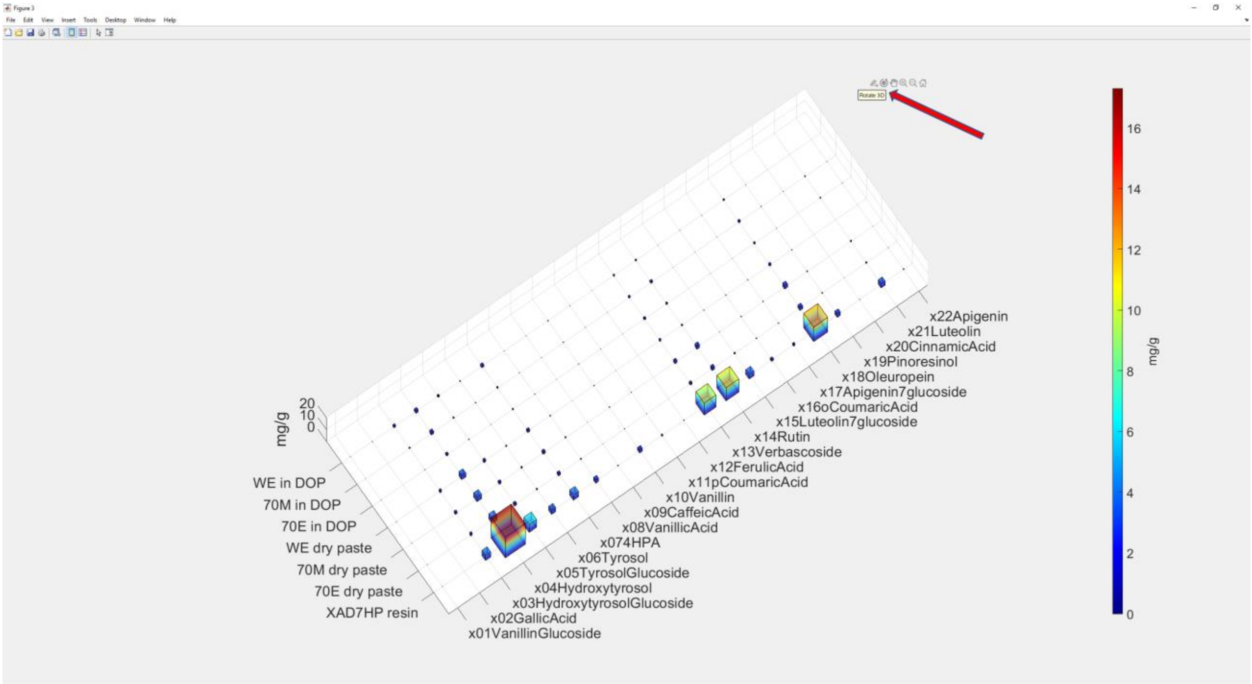
x22Apigenin
x21Luteolin
x20CinnamicAcid
x19Pinoresinol
x18Oleuropein
x17Apigenin7glucoside
x16oCoumaricAcid
x15Luteolin7glucoside
x14Rutin
x13Verbascoside
x12FerulicAcid
x11pCoumaricAcid
x10Vanillin
x09CaffeicAcid
x08VanillicAcid
x074HPA
x06Tyrosol
x05TyrosolGlucoside
x04Hydroxytyrosol
x03HydroxytyrosolGlucoside
x02GallicAcid
x01VanillinGlucoside
WE in DOP
70M in DOP
70E in DOP
WE dry paste
70M dry paste
70E dry paste
XAD7HP resin
mg/g
20
10
0
mg/g
16
14
12
10
8
6
4
2
0

(a)

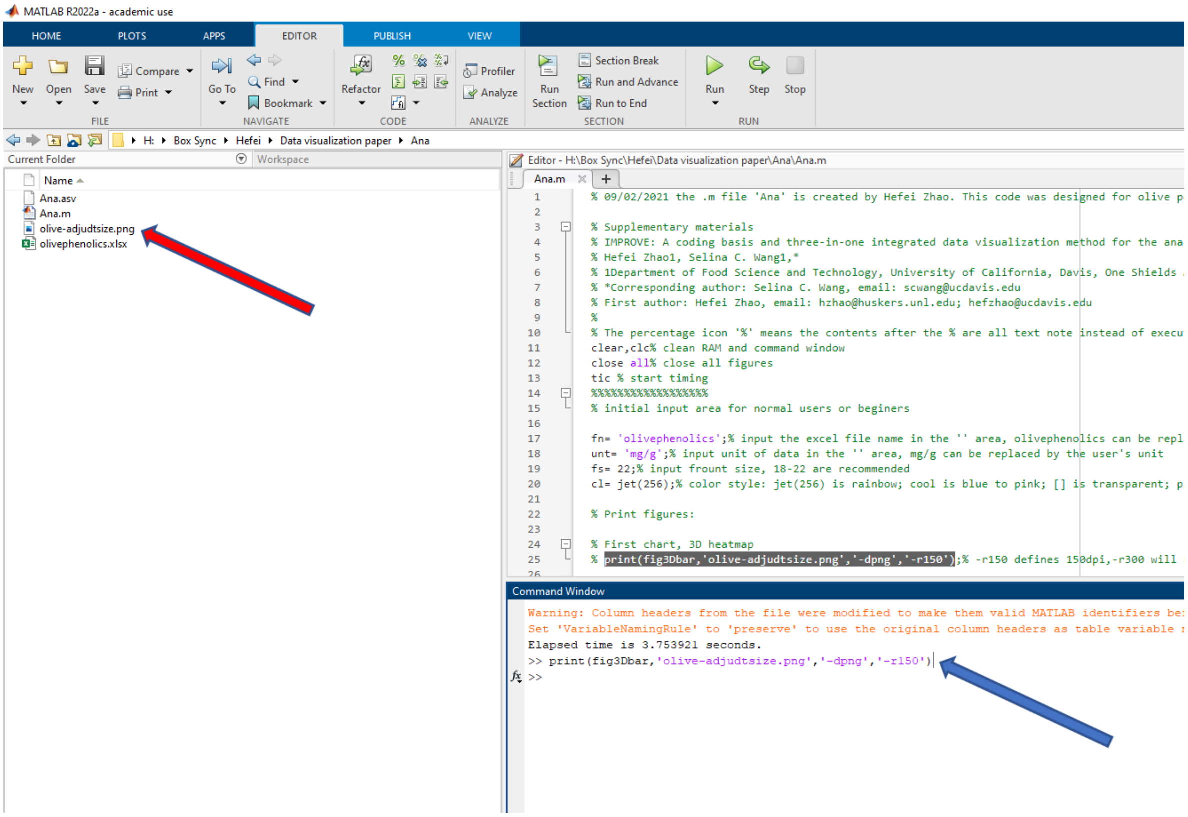
MATLAB R2022a - academic use
HOME
PLOTS
APPS
EDITOR
PUBLISH
VIEW
Current Folder
Workspace
Name
Ana.asv
Ana.m
olive-adjudtsize.png
olivephenolics.xlsx
Editor - H:\Box Sync\Hefei\Data visualization paper\Ana\Ana.m
Ana.m
% 09/02/2021 the .m file 'Ana' is created by Hefei Zhao. This code was designed for olive p
% Supplementary materials
% IMPROVE: A coding basis and three-in-one integrated data visualization method for the ana
% Hefei Zhao1, Selina C. Wang1,*
% 1Department of Food Science and Technology, University of California, Davis, One Shields
% *Corresponding author: Selina C. Wang, email: scwang@ucdavis.edu
% First author: Hefei Zhao, email: hzhao@huskers.unl.edu; hefzhao@ucdavis.edu
% The percentage icon '%' means the contents after the % are all text note instead of execu
clear,clc% clean RAM and command window
close all% close all figures
tic % start timing
% initial input area for normal users or beginers
fn= 'olivephenolics';% input the excel file name in the '' area, olivephenolics can be repl
unt= 'mg/g';% input unit of data in the '' area, mg/g can be replaced by the user's unit
fs= 22;% input frount size, 18-22 are recommended
cl= jet(256);% color style: jet(256) is rainbow; cool is blue to pink; [] is transparent; p
% Print figures:
% First chart, 3D heatmap
% print(fig3Dbar,'olive-adjudtsize.png','-dpng','-r150');% -r150 defines 150dpi,-r300 will
Command Window
Warning: Column headers from the file were modified to make them valid MATLAB identifiers be
Set 'VariableNamingRule' to 'preserve' to use the original column headers as table variable
Elapsed time is 3.753921 seconds.
>> print(fig3Dbar,'olive-adjudtsize.png','-dpng','-r150')
>>

(b)

Figure S3. By clicking the rotation icon (red arrow) in (a), figures will pop up, then (b) copy print(fig3Dbar,'olive-adjudtsize.png','-dpng','-r150'), then paste in the command window (the blue arrow), then press keyboard 'Enter', a '.png' file at 150 dpi will show up in the 'current folder' (red arrow)

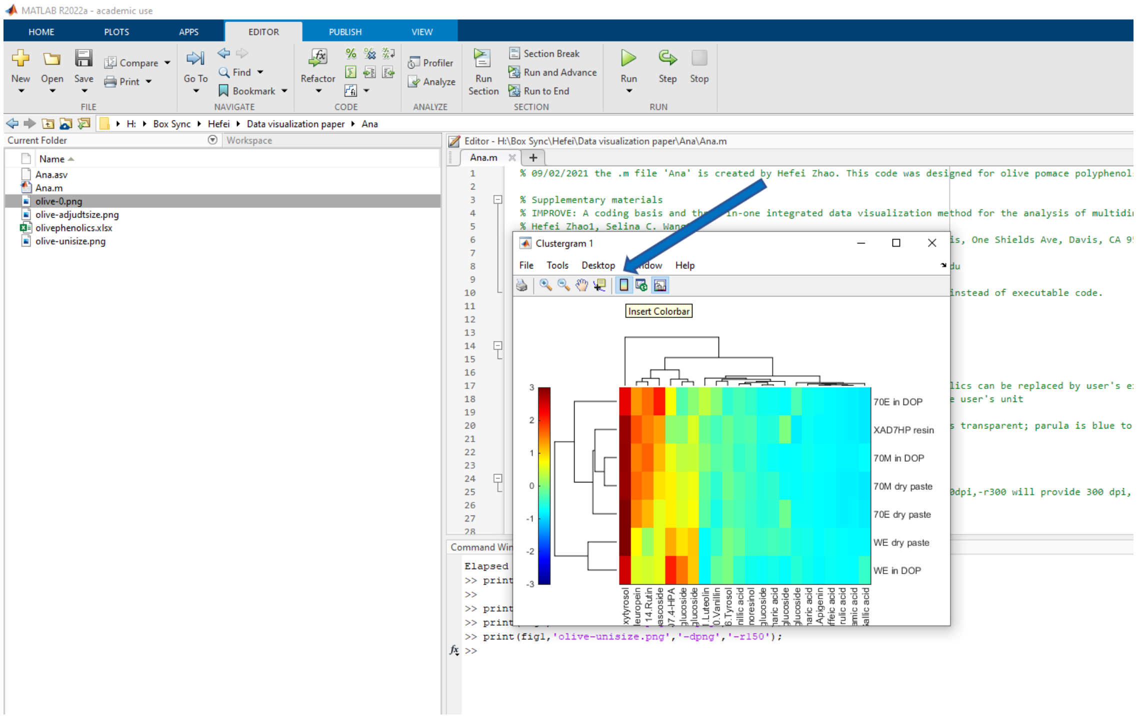

(a)

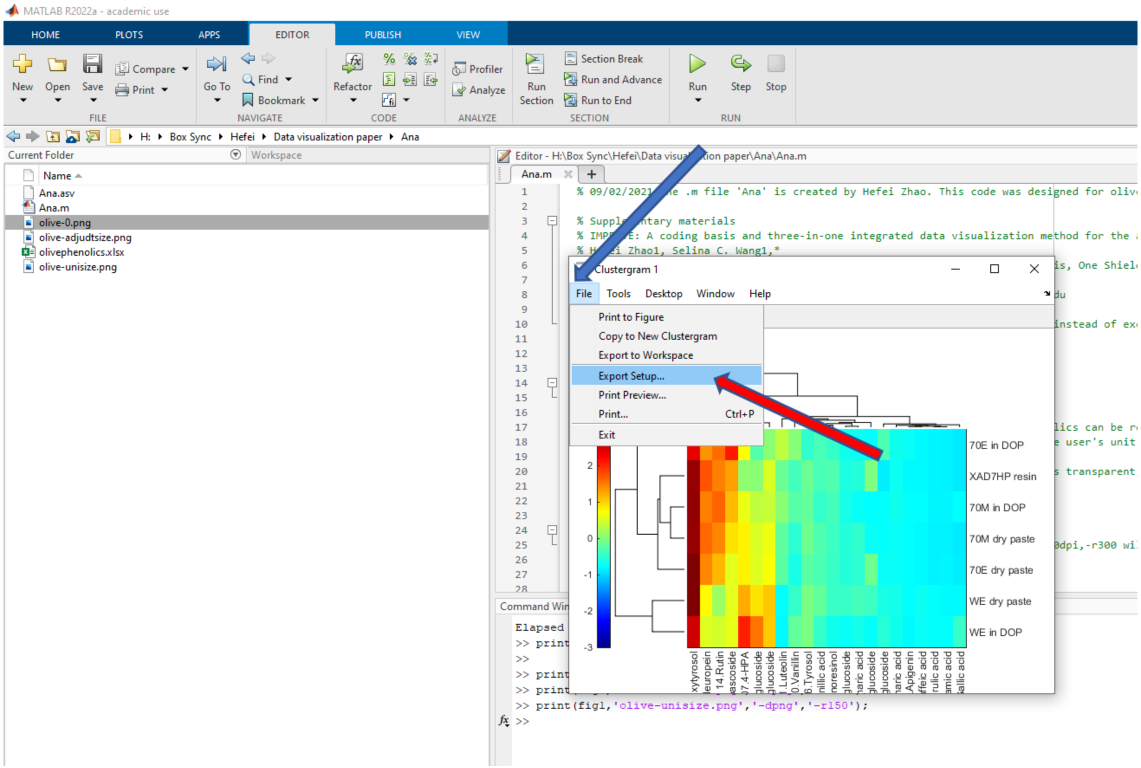

(b)

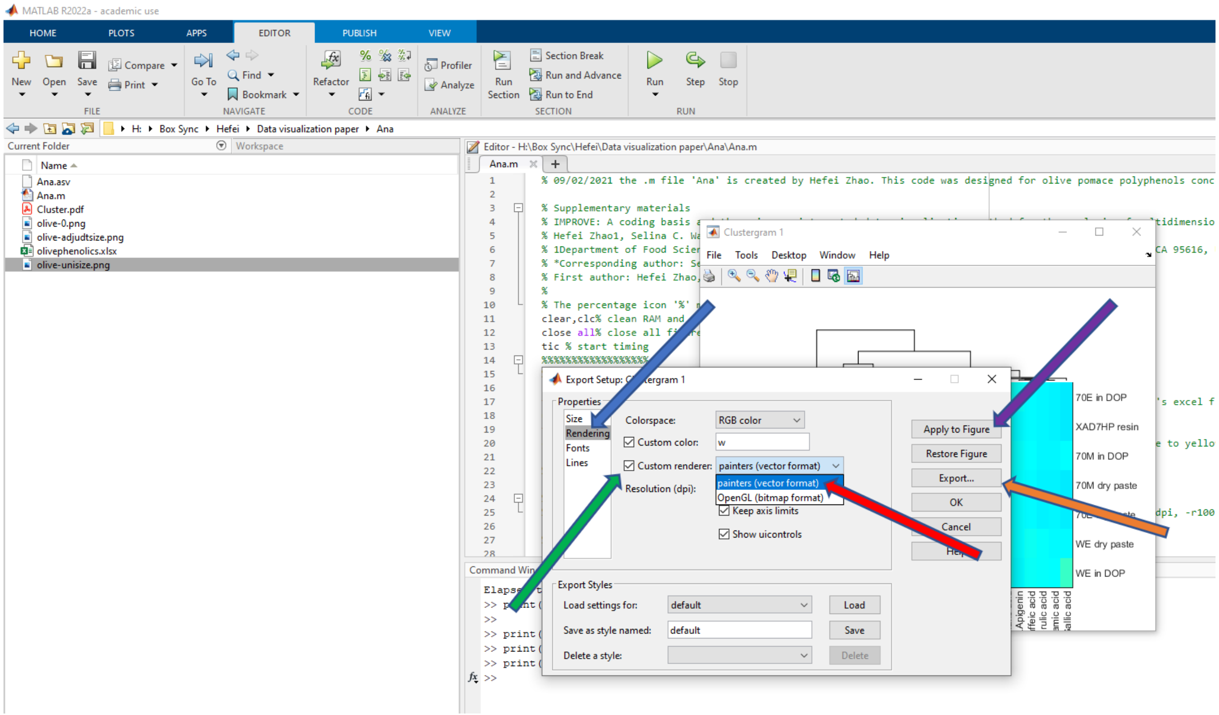
MATLAB R2022a - academic use
HOME
PLOTS
APPS
EDITOR
PUBLISH
VIEW
Current Folder
Workspace
Ana.asv
Ana.m
Cluster.pdf
olive-0.png
olive-adjudtsize.png
olivephenolics.xlsx
olive-unisize.png
Editor - H:\Box Sync\Hefei\Data visualization paper\Ana\Ana.m
% 09/02/2021 the .m file 'Ana' is created by Hefei Zhao. This code was designed for olive pomace polyphenols conc
% Supplementary materials
% Hefei Zhao1, Selina C. Wa
% 1Department of Food Scie
% *Corresponding author: Se
% First author: Hefei Zhao,
clear,clc% clean RAM and
close all% close all fi
tic % start timing
Clustergram 1
File
Tools
Desktop
Window
Help
Export Setup: Clustergram 1
Properties
Size
Rendering
Fonts
Lines
Colorspace:
RGB color
Custom color:
w
Custom renderer:
painters (vector format)
OpenGL (bitmap format)
Resolution (dpi):
Keep axis limits
Show uicontrols
Apply to Figure
Restore Figure
Export...
OK
Cancel
Help
Export Styles
Load settings for:
default
Load
Save as style named:
Save
Delete a style:
Delete
70E in DOP
XAD7HP resin
70M in DOP
70M dry paste
WE dry paste
WE in DOP
Command Window

(c)

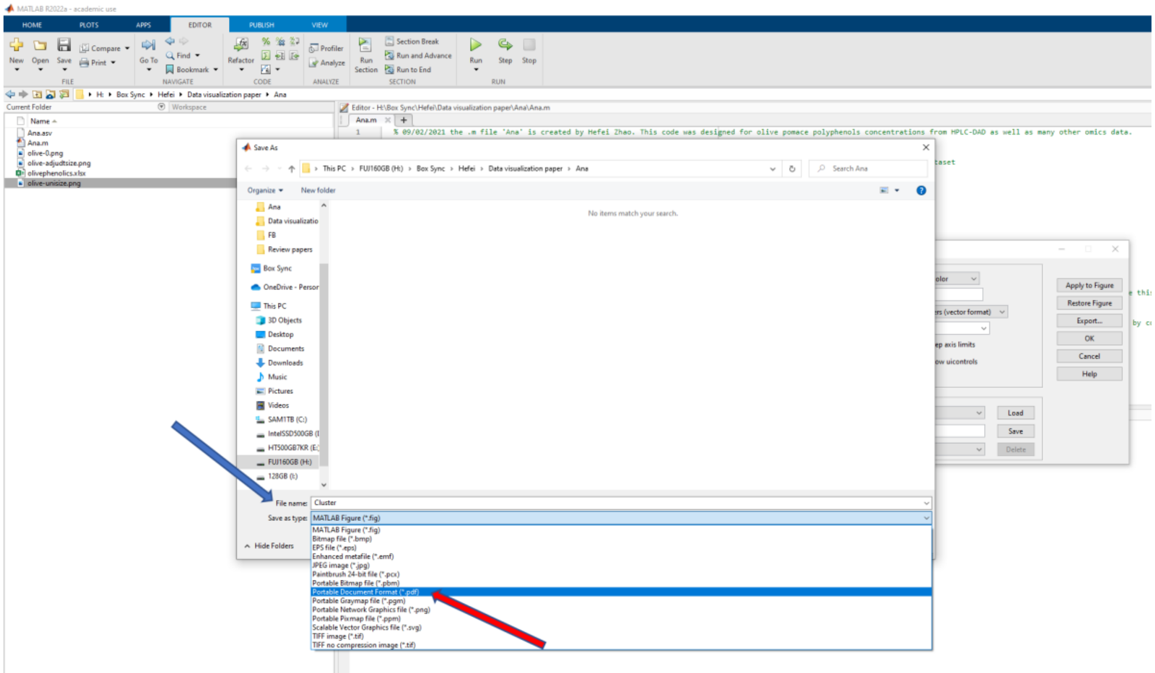
Save As
This PC
Box Sync
Hefei
Data visualization paper
Ana
Organize
New folder
No items match your search.
File name:
Cluster
Save as type:
MATLAB Figure (*.fig)
Bitmap file (*.bmp)
EPS file (*.eps)
Enhanced metafile (*.emf)
JPEG image (*.jpg)
Paintbrush 24-bit file (*.pcx)
Portable Bitmap file (*.pbm)
Portable Document Format (*.pdf)
Portable Graymap file (*.pgm)
Portable Network Graphics file (*.png)
Portable Pixmap file (*.ppm)
Scalable Vector Graphics file (*.svg)
TIFF image (*.tif)
TIFF no compression image (*.tif)
Hide Folders

(d)

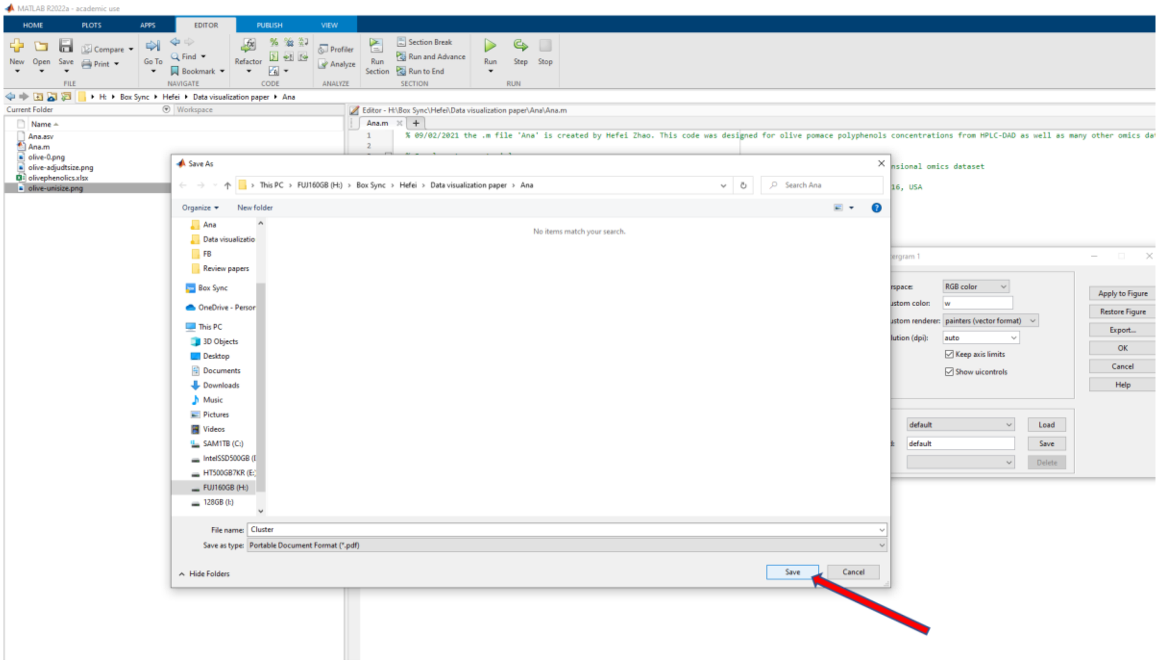

(e)

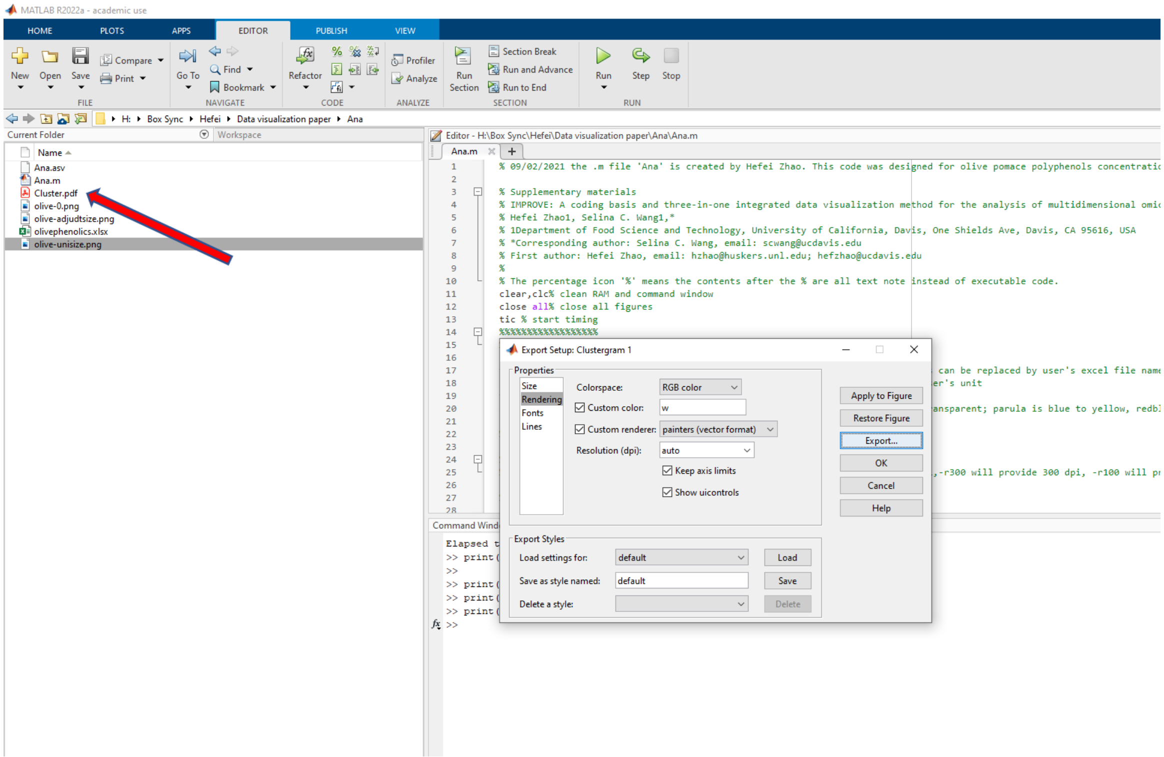

(f)

Figure S4. Export heatmap cluster chart to a ‘.pdf’ file, (a) on the ‘Clustergram 1’ window, click ‘Insert Colorbar’, (b) on the ‘Clustergram 1’ window, click ‘File’, then click ‘Export Setup’, (c) click ‘Rendering’, select ‘Custom rendering’ as ‘Painters (vector format)’, very important for high-resolution output!!!, click ‘Apply to Figure’, then click ‘Export’, (d) input ‘File name’ as ‘Cluster’, select ‘Save as type’ the ‘Portable Document Format (*.pdf)’, (e)

click ‘Save’, (f) a ‘Cluster.pdf’ file will show up in the ‘Current Folder’. Then open the ‘.pdf’ file for ‘print screen’ a high-resolution figure.

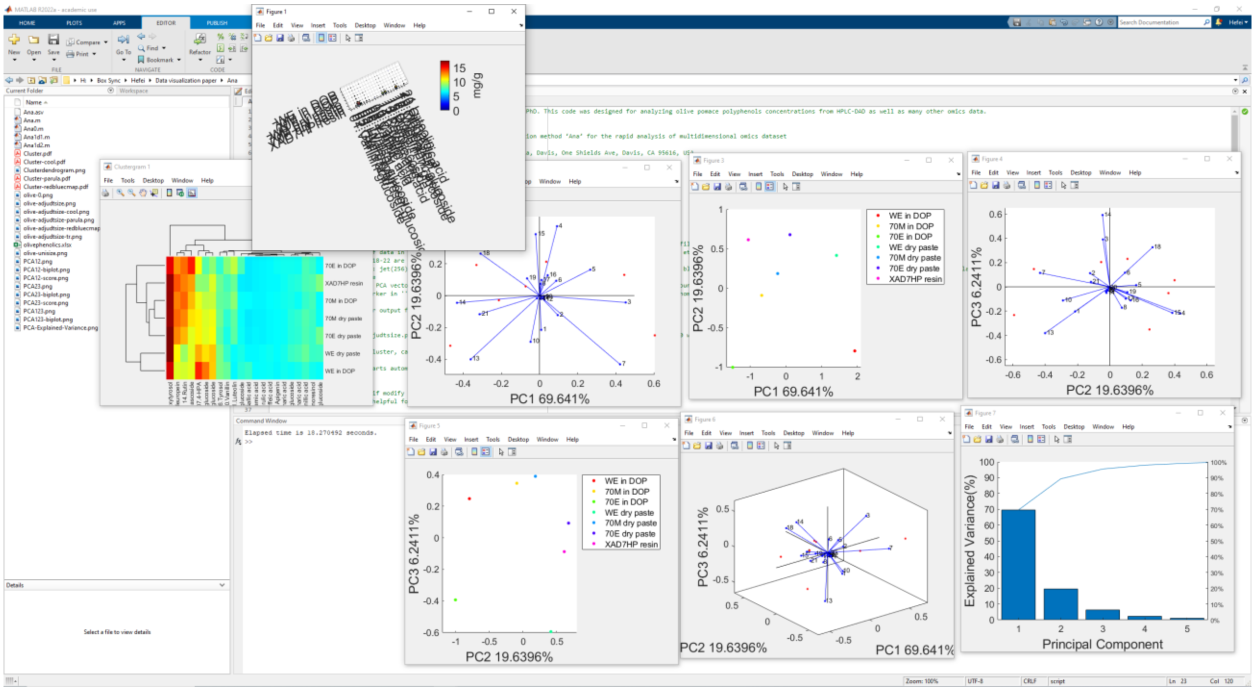

(a)

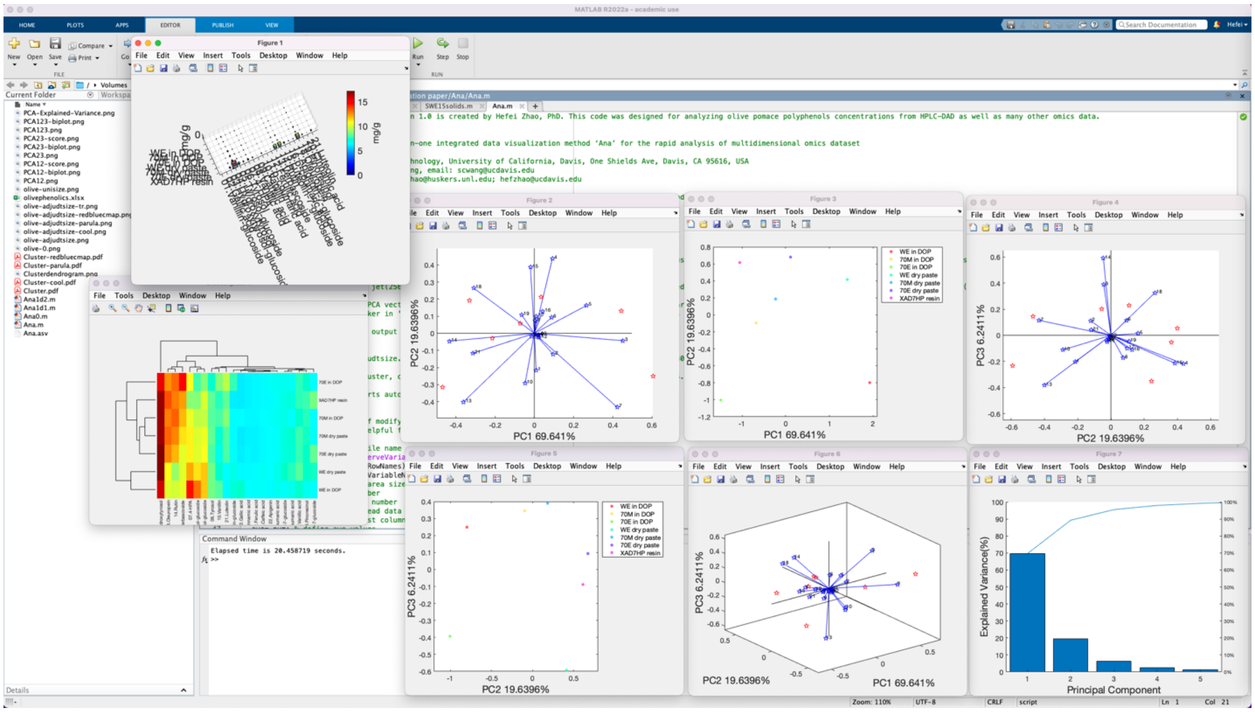

(b)

Figure S5. Final outcomes of the program running. (a) data analyzed and output six figures in 18 seconds in the PC with Windows 10 system, (b) data analyzed and output six figures in 20 seconds in MacOS Monterey system.

S. Tables

Table S1. Phenolic compound data of olive pomace extract, data from our previous publication [2].

NAME	01.Vanillin-glucoside	02.Gallic acid	03.Hydroxytyrosol-glucoside	04.Hydroxytyrosol	05.Tyrosol-glucoside	06.Tyrosol	07.4-HPA	08.Vanillic acid	09.Caffeic acid	10.Vanillin	11.p-coumaric acid	12.Ferulic acid	13.Verbascoside	14. Rutin	15.Luteolin-7-glucoside	16.o-coumaric acid	17.Apigenin-7-glucoside	18.Oleuropein	19.Pinoresinol	20.Cinnamic acid	21.Luteolin	22.Apigenin
WE in DOP	0.165	0.223	1.407	1.978	1.096	0.460	1.700	0.203	0.050	0.371	0.084	0.047	0.833	0.770	0.042	0.101	0.055	0.811	0.084	0.027	0.010	0.007
70M in DOP	0.054	0.045	0.657	2.017	0.679	0.365	0.800	0.208	0.044	0.329	0.097	0.023	1.074	1.360	0.042	0.070	0.121	1.270	0.257	0.019	0.487	0.062
70E in DOP	0.225	0.000	0.250	1.356	0.384	0.162	0.660	0.223	0.039	0.375	0.086	0.029	1.232	1.031	0.042	0.070	0.088	0.930	0.175	0.013	0.515	0.066
WE dry paste	0.151	0.007	1.423	3.508	1.555	0.624	1.691	0.509	0.073	0.285	0.131	0.043	1.135	0.791	0.312	0.352	0.293	1.298	0.300	0.012	0.041	0.030
70M dry paste	0.155	0.008	1.475	3.880	1.581	0.811	1.755	0.609	0.102	0.385	0.168	0.046	1.858	2.409	0.175	0.416	0.341	2.609	0.478	0.043	0.714	0.111
70E dry paste	0.152	0.010	1.480	4.219	1.639	0.666	1.742	0.585	0.091	0.269	0.157	0.047	1.507	2.108	0.785	0.369	0.336	2.393	0.461	0.063	0.678	0.107
XAD7HP resin	0.187	-0.018	4.423	17.298	6.519	3.514	4.450	2.530	0.420	2.439	0.884	0.326	10.159	11.048	4.086	1.562	1.345	12.231	2.775	0.205	3.515	0.469

Note: WE, water extract; 70M, 70% methanol extract; 70E, 70% ethanol extract; XAD7HP resin, XAD7HP resin purified extract

S. References

1 Zhao, H.; Wang, C.S. A 3in1 Omics Data Visualization and Analytical Method—File Exchange—MATLAB Central. Available online: https://www.mathworks.com/matlabcentral/fileexchange/117370-a-3in1-omics-data-visualization-and-analytical-method (accessed on 8 September 2022).

2 Zhao, H.; Avena-Bustillos, R.J.; Wang, S.C. Extraction, Purification and In Vitro Antioxidant Activity Evaluation of Phenolic Compounds in California Olive Pomace. Foods 2022, 11, 174.
